# Removal of the Inferior Maxilla for Malignant Epulis: A Clinical Lecture in Cook Co. Hospital, by Prof. J. W. Freer

**Published:** 1872-06-01

**Authors:** L. Curtis


### Clinical Reports.

### REMOVAL OF THE INFERIOR MAXILLA FOR MALIGNANT EPULIS: A CLINICAL LECTURE IN COOK CO. HOSPITAL, BY PROF. J. W. FREER.

REPORTED BY L. CURTIS, M.D.

Professors Gunn, Powell, Lyman, Dr. Owens and others, were present as assistants. Every precaution had been taken to ensure the success of the operation. A charcoal furnace was placed within the amphitheater, in which were heating cauterizing irons for use in case of troublesome hemorrhage.

After all preparations had been made, the patient, a man of about sixty years of age, though appearing much older, was led in. He was emaciated and pale, and appeared exhausted by his exertions in walking into the room. A mottled red and gray tumor projected from the mouth, across which the distended lower lip was tightly drawn. On the entrance of the patient, the Professor commenced his remarks:

"*Gentlemen:* We have here a peculiar case. One would think on superficial examination that it was a disease of the tongue. I thought so myself when I first saw it. But if we look into the mouth, we shall find the tongue to be intact. It is even tipped a little back by the crowding of the tumor. This tumor commenced four months ago as a little spot on the gums, and below the teeth. It is called epulis. An ordinary epulis generally does not involve more than three or four teeth. The periosteum is first involved, the bone secondarily. The gum has a spongy appearance, and rises up between the teeth, crowding them out of their sockets, and they appear on the top of the tumor. If the tumor is removed with a part of the alveolar process, the disease is arrested. Anyone can do it, almost.

"Sometimes, however, it takes on the character of a malignant growth. Paget speaks of it as recurrent fibroid. In Erickson's Surgery it is called malignant epulis. This is the kind of tumor we have here. It is endangering the patient's life, and has already occasioned severe dyspnœa. If left to itself, he could only live a few days. The operation for its removal is a severe and dangerous one; but I am ably supported, and the patient consents. I have told him the danger; but he says that if he dies it will be over. I would not consent to operate, however, if not so ably supported. We do not intend to let the patient die under the knife.

"The dangers are manifold, the principal of which is hemorrhage. The tumor has grown so fast that it is exceedingly vascular. We shall remove the lower jaw. If we do this, we encounter another danger. The muscular connections of the tongue, the genio-hyo-glossus, etc., are severed, and the tongue is liable to drop back, close the epiglottis, and cause the death of the patient by suffocation. Accordingly, we pass a ligature through the tongue before commencing. Operations of this kind cannot be performed rapidly; we must proceed with caution, and arrest hemorrhage as we go."

The patient was then put under the influence of ether. Some difficulty was experienced in passing a ligature through the tongue; it was accordingly seized with a tenaculum.

An incision was made, commencing just below the ear on the right side, following the lower border of the jaw; the facial artery was divided and tied; the face was then turned in an opposite direction, and a similar incision was made on the left side; the masseter muscle was divided; the chain saw, by means of a stout, well-curved needle, was passed under the ramus of the jaw; and the ramus was sawed off just below the articulation.

The Doctor remarked that he had taken special pains to avoid the facial nerve, an important point, mention of which was neglected in the books. The books say, Make a free incision as high as the articulation. He would have carried the incision no higher, even if he had been going to disarticulate the jaw, for it was not necessary.

The incision was then carried around the chin, and was met by another incision, dividing the lip perpendicularly; the left cheek was then dissected from the bone; the tongue was reached, and a ligature was passed through its tip; the face was then turned to the other side; the masseter muscle was divided; the chain saw was passed under the ramus; and the bone was sawn through as it had been done on the previous side.

An attempt was made to dissect the jaw out entire; but the fragile bone broke near the symphisis, and the pieces were taken out separately. On the removal of the pieces of bone, the tissue under the tongue was found to be much indurated. In the attempt to remove some of this indurated tissue, quite a large artery was divided and ligated. It was found impossible to remove all of this indurated tissue, and the actual cautery was applied. The tongue was fastened to one of the incisor teeth by means of the ligature; the divided lip was united by hare-lip-pins; and the remainder of the wound by sutures.

The bone was found, on examination, to have been involved in the tumor, and to have been almost completely eaten away by it, from the right of the symphisis nearly to the angle of the jaw. The disease extended also along the alveolar process, lifting the teeth up and loosening them, nearly to the angle on the right side.

In conclusion, the Professor remarked that if it were not for the malignant disease, the operation would not occasion much disfigurement to the patient. The bone would be replaced by tough fibrous tissue, which would preserve the form of the face very well.

With regard to the tongue, at present it would be unsafe to leave it without its being secured; but if the patient lived, the muscles would form attachments, and it could be released, and he could use it in a few days.

The patient reacted well, experiencing but little if any more shock than from an ordinary thigh amputation in the lower third.

